# Management of prostacyclin side effects in adult patients with pulmonary arterial hypertension

**DOI:** 10.1177/2045893217719250

**Published:** 2017-07-11

**Authors:** Martha Kingman, Christine Archer-Chicko, Mary Bartlett, Joy Beckmann, Robin Hohsfield, Sandra Lombardi

**Affiliations:** 1 University of Texas Southwestern Medical Center at Dallas, Dallas, TX, USA; 2 Penn Presbyterian Medical Center, Philadelphia, PA, USA; 3 Winthrop University Hospital, Mineola, NY, USA; 4 Harbor UCLA Medical Center, Torrance, CA, USA; 5 University of Colorado Health, Denver, CO, USA; 6 University of California, San Diego, La Jolla, CA, USA

**Keywords:** adverse events, prostacyclin therapy, pulmonary arterial hypertension, pulmonary hypertension

## Abstract

Therapies that target the prostacyclin pathway are considered effective, yet are complex to dose and may cause dose-limiting side effects for patients with pulmonary arterial hypertension (PAH). Careful side effect management and the ability to discern side effects from worsening disease are essential in order for patients to continue, and benefit from, prostacyclin therapy. This manuscript was developed through a collaborative effort of allied health providers with extensive experience in managing patients with PAH who are treated with medications that target the prostacyclin pathway. This article provides an overview of individual prostacyclin pathway therapies approved in the United States, side effects most commonly associated with these therapies, and practical suggestions for side effect management. Most patients will experience significant side effects on prostacyclin therapy. Creating a proactive and careful side effect management program will increase the likelihood that patients are able to stay on therapy and receive the benefits afforded by prostacyclin therapy.

## Introduction

Pulmonary arterial hypertension (PAH) is a rare disease characterized by vascular proliferation and vasoconstriction of the pulmonary arterial bed, leading to increasing pulmonary vascular resistance (PVR) over time.^1^ The right ventricle will begin to enlarge with the increased workload required to pump blood through narrowed pulmonary arteries. If left untreated, the right ventricle will ultimately fail, leading to death.^2,3^

PAH can only be diagnosed by right heart catheterization (RHC) and is defined as a mean pulmonary capillary pressure ≥25mmHg, pulmonary capillary wedge pressure ≤15 mmHg, and a PVR >3 Wood Units.^1,4,5^ The broader term “pulmonary hypertension” is categorized into five World Health Organization (WHO) groups according to clinical presentation, pathological findings, and hemodynamic characteristics.^5^ WHO Group 1 includes patients with PAH, which is the focus of this manuscript and is the only cohort of patients for which prostacyclin therapies are approved.

Three pathways, endothelin, nitric oxide, and prostacyclin, are involved in PAH pathophysiology.^6^ Fourteen medications targeting these pathways are approved by the Food and Drug Administration (FDA) for treatment of PAH, seven of which target the prostacyclin pathway.^1^ Due to the high level of evidence of efficacy, prostacyclin is generally thought to be the standard of care for PH patients with advanced disease.^1^

In patients with PAH, prostacyclin synthase is reduced leading to inadequate production of prostaglandin I_2_ (PGI_2_), or prostacyclin. This prostacyclin deficit contributes to the vasoconstriction, vascular proliferation, and platelet aggregation seen in patients with PAH. Prostacyclin's main target, the IP receptor, is expressed in the pulmonary vascular smooth muscle cell layer.^7^ Activation of the IP receptor stimulates adenylyl cyclase leading to increased cyclic adenosine monophosphate, which increases protein kinase A activity. This leads to vasodilation, antithrombosis, and anti-proliferative effects on vessel walls.^6–13^ Synthetic prostacyclin analogues were developed to replace the endogenous prostacyclin deficiency in patients with PAH. The parental PGI_2_ analogues tend to be more complex to administer and have more side effects than other classes of drugs used to treat PAH; therefore, PGI_2_ analogues are often reserved for patients with more advanced disease.^11^

Although adherence to treatments in PAH has resulted in marked improvement in symptoms and disease progression, side effects often impact quality of life in patients^8,10,11^ with PAH and can lead to discontinuation. This article will review the management of the common side effects of FDA-approved medications that target the prostacyclin pathway (Table 1).

**Table 1.** Common side effects of medications that target the prostacyclin pathway.

Medications	Route	Related to the administration routes	Related to the pharmacological action
Epoprostenol	Continuous intravenous infusion	Catheter-related infections, sepsis, thromboembolic event, bleeding, drug-delivery system malfunction	Jaw pain, diarrhea, flushing, headaches, nausea, vomiting
Iloprost	Inhaled	Cough, throat irritation	Flushing, jaw pain, headaches, hypotension, body aches, nausea, diarrhea, dizziness
Treprostinil	Oral	Pill shell may not be absorbed and may be visibly excreted in the feces	Headaches, flushing, nausea, diarrhea, jaw pain, vomiting, extremity pain
	Continuous subcutaneous infusion	Infusion site pain, site reaction, and site abscess	Diarrhea, jaw pain, flushing, nausea, rash, dizziness, vomiting, headaches, flushing
	Continuous intravenous infusion	Catheter-related infections, thromboembolic event, drug-delivery system malfunction	Extremity pain, headaches, diarrhea, jaw pain, nausea, fatigue, loose stools, vomiting, dizziness, dyspnea, flushing, palpitations, peripheral edema
	Inhaled	Cough, throat irritation,	Headaches, nausea, flushing, diarrhea, dizziness
		Pharyngolaryngeal pain	Dizziness
Selexipag	Oral	N/A	Headache, diarrhea, jaw pain, nausea, myalgia, vomiting, pain in extremity, flushing

N/A, not applicable.

## Epoprostenol

Epoprostenol sodium (Flolan®)^14^ was the first targeted therapy for the treatment of PAH and was approved in the US in 1995. This formulation required the use of ice packs surrounding the medication, to maintain stability. Epoprostenol for injection (Veletri®)^15^ was approved in 2008, which was developed for room-temperature stability.

Both formulations of epoprostenol have a short half-life of 2–3 min and require continuous administration with an ambulatory, infusion pump through a tunneled central venous catheter or a peripherally inserted central catheter (PICC) line.

Prostacyclin medications are the only therapies with proven survival benefit.^16^ A meta-analysis of epoprostenol studies revealed a 70% mortality risk reduction.^17^ Due to its superior efficacy, epoprostenol is considered first-line therapy for patients with PAH who have advanced disease and/or functional class (FC) IV symptoms.^1^ Common side effects of epoprostenol include nausea, vomiting, diarrhea, headache, hypotension, flushing, dizziness, jaw pain, and musculoskeletal pain (Table 1).^15^

## Iloprost

Inhaled iloprost (Ventavis®)^18^ is a synthetic analogue of prostacyclin PGI_2_, approved in the US in 2004. Iloprost is inhaled directly into the lungs, and at the level of the alveoli, crosses over to the intra-acinar pulmonary arteries causing vasodilaton.^19^ The advantages of inhaled iloprost include ease of administration and no risk for central line sepsis, which can occur with therapies that require central venous access. Improved ventilation-perfusion matching is another advantage, as the medication is inhaled only into well-ventilated lung alveoli.^19^

Iloprost has a short half-life of 20–25 min and therefore requires six to nine treatments per day, equating to dosing approximately every 2 h while awake. Administration of iloprost requires a specially designed adaptive aerosol delivery device, which matches the patient's individual breathing pattern.^19^ Common side effects include vasodilation (flushing), cough, headache, jaw pain, nausea, hypotension, and vomiting (Table 1).^18^

## Treprostinil

Treprostinil is a tricyclic benzidine prostacyclin available in several formulations: intravenously and subcutaneously (Remodulin®),^20^ inhaled (Tyvaso®),^21^ and extended-release tablets for oral dosing (Orenitram®).^22^

Subcutaneous (SQ) treprostinil was approved in 2002 in the US. This formulation has a 4-h half-life and offers patients the benefits of prostacyclin therapy without an invasive central venous catheter and the associated risk of central line blood stream infections. SQ treprostinil is administered through a small catheter inserted under the skin, which is attached to a small pump. Common side effects reported in clinical studies include infusion site pain and reaction, headache, diarrhea, nausea, jaw pain, vasodilation, and hypotension (Table 1).^20^

Intravenous (IV) treprostinil was approved by the FDA in 2004. Similar to IV epoprostenol, IV treprostinil is administered via an ambulatory infusion pump through a central venous catheter. IV treprostinil also has a half-life of approximately 4 h. Most common side effects for IV treprostinil are the same as SQ treprostinil, with the exception of site pain and reaction that is not seen with IV administration (Table 1).^20^ A notable complication of IV treprostinil is the concern for increased risk of bloodstream infection with gram-negative organisms.^23^

Inhaled treprostinil was approved in the US in 2009.^21^ This formulation has a half-life of 3–4 h and is administered as an inhaled treatment four times a day using a battery powered device. Inhaled treprostinil is sometimes added to oral therapy for patients who may not be candidates for parenteral therapy.^24^ Common side effects include cough, headache, nausea, dizziness, flushing, throat irritation, pharynlaryngeal pain, and diarrhea (Table 1).^21^

The oral formulation of treprostinil was approved in the US in 2013.^22^ Treprostinil diolamine was developed as a sustained release osmotic capsule allowing for two or three times per day dosing. The half-life of oral treprostinil is about 1–1.5 h.^25^ Oral treprostinil should be taken with food and should be swallowed whole, not crushed, split, or chewed.^22^ Recommended starting dose is 0.25 mg twice daily with food, taken approximately 12 h apart or 0.125 mg three times daily with food, taken approximately 8 h apart.^22^ The dose should be increased at increments of 0.25 or 0.5 mg twice daily or 0.125 mg three times daily every three to four days to the highest tolerated dose.^22^ If dose increments are not tolerated, consider titrating slower.^22^ Common side effects include headache, diarrhea, nausea, flushing, jaw pain, and leg pain (Table 1).^22^

## Selexipag

Selexipag (Uptravi®),^26^ approved in the US in 2015, is an orally available selective non-prostacyclin that is an agonist to the prostacyclin IP receptor. Selexipag is hydrolyzed to an active metabolite which is approximately 37-fold more potent than selexipag.^26^ Both selexipag and its metabolite are highly selective for the IP receptor, and the metabolite has a 7.9-h half-life, which allows for twice daily dosing.^27^ Common side effects include headache, diarrhea, jaw pain, nausea, myalgia, vomiting, pain in extremity, and flushing (Table 1).^26^

## Managing side effects of prostacyclin pathway therapies

In order to maximize the benefit of prostacyclin pathway therapies, healthcare providers must identify, accurately assess, and appropriately manage prostacyclin side effects which otherwise may lead to discontinuation of therapy.

Due to the difficult side effect profile, prostacyclin pathway therapies are initiated at low doses and up-titrated slowly, as tolerated, until a maintenance dose is achieved.^19^ In general, side effects are more common during the titration phase than in the maintenance phase.^26,27^ Patients should be educated to expect some side effects, which vary from patient to patient. Advances in treatment options have shown improvements in physical functioning and survival.^28^ Despite considerable side effects, patients receiving prostacyclin pathway therapies have shown improvement in their quality of life.^11^

## Common side effects with prostacyclin pathway therapies

**Fig. 1.**
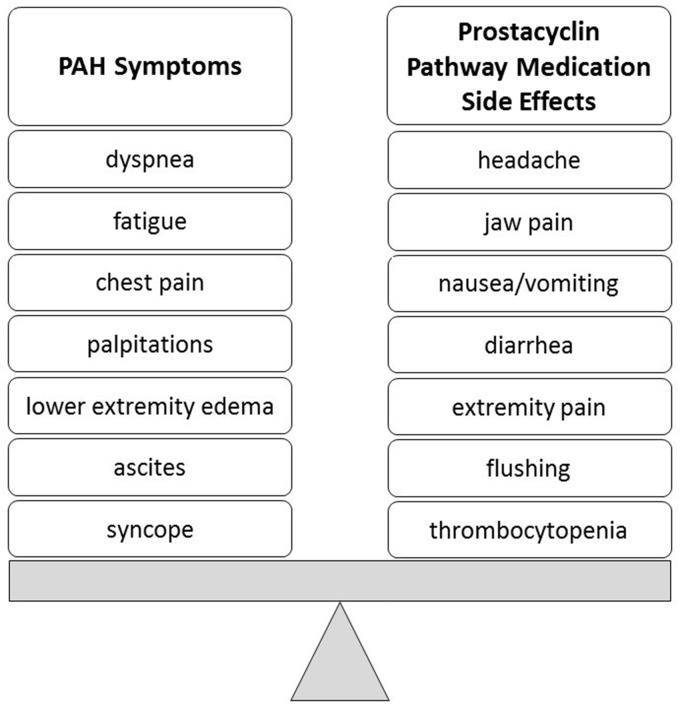
Balancing PAH symptoms versus prostacyclin pathway therapy side effects. PAH, pulmonary arterial hypertension.

Recognizing the difference between side effects of prostacyclin pathway therapies and the signs and symptoms of the disease such as worsening right heart failure (Fig. 1) is an important first step. The common side effects (Table 1) of prostacyclin pathway therapies are discussed below, along with the healthcare provider management suggestions for each (Table 2).

**Table 2.** Prostacyclin pathway side effects: interventions and considerations.

Side effect	Intervention	Consideration/rationale
**Pain management**	Non-pharmacologic: heating pad, massage, acupuncture, acupressure, relaxation techniques	First-line options to relieve pain
	Pharmacologic: acetaminophen, ibuprofen (if not contraindicated), gabapentin, pregabalin, tramadol, narcotics-hydrocodone, fentanyl, or codeine on a case-by-case basis	Second-line option to relive pain
	Lowering the dose of prostacyclin or switching to a different prostacyclin therapy	Dose-limiting side effect
	Refractory pain should be referred to pain management or palliative care	Pain management is better trained and equipped to manage severe long-term pain
**Leg pain**	Screen for iron deficiency; Gabapentin may be a more successful analgesic for leg pain	Decreased RBC oxygen carrying capacity, with reduced circulation in lower extremities
**Jaw pain**	Usually no interventions needed; reassure patient that this will get better with time	Not a dose-limiting side effect, loss of jaw pain may indicate need for up-titration
	Take slow bites or sips of water, suck on saltine cracker or hard candy, chew gum before eating	Jaw pain is intermittent and generally occurs with first bite of the meal
**Site pain**	Ice, warm bath with Epsom salt, aloe vera gel, arnica oil, capsaicin cream	First-line options to relieve site reaction or pain
	Anesthetic agents (lidocaine 5% patches, lidocaine/prilocaine cream, calamine lotion/cream [pramoxine], menthol/methyl salicylate cream)	Analgesic and anti-pruritic effects; used during initiation phase of new subcutaneous site
	Vasoconstrictive agents (hemorrhoid ointment)	Anti-pruritic and relieves discomfort
	Corticosteroids (hydrocortisone cream, triamcinolone acetonide, fluticasone propionate nasal spray, clobetasol propionate cream)	Anti-pruritic and anti-inflammatory; relieves erythema and discomfort
	Calcineurin inhibitors (pimecrolimus cream)	Second-line option in patients who have failed to respond adequately to other topical prescription treatments
	Histamine H1 receptor antagonists (diphenhydramine HCl, topical)	Anti-pruritic and anti-inflammatory
	Histamine H1 and H2 antagonist (doxepin cream)	Anti-pruritic and anti-inflammatory
	PLO gel compounds, microemulsion-based gel that has been used to deliver different types of pain drugs topically and transdermally	Anti-pruritic and anti-inflammatory; extensively used (for current and old sites), often as first-line option
	Non-opioid analgesics (ibuprofen, acetaminophen)	Non-opioid analgesics, first-line options
	GABA analogs (gabapentin, pregabalin)	Second-line option after non-opioid analgesics
	First-generation anti-histamine (hydroxyzine pamoate)	Anti-pruritic and anti-inflammatory (for severe cases)
	Histamine H1 receptor antagonists (loratadine, fexofenadine HCl, cetirizine HCl, ranitidine HCl, famotidine)	Anti-pruritic and anti-inflammatory
	Opioid analgesics (tramadol HCl, fentanyl patch, hydrocodone with acetaminophen	For severe pain
	Antidepressant (amitriptyline HCl)	Used as analgesic for chronic pain
**Headache**	Pre-treat before oral or inhaled doses or prior to up-titration of parenteral prostacyclin	Used to prevent headache or lessen severity
**Mild/moderate**	Acetaminophen	Use smallest amount for the shortest amount of time and monitor LFTs
	Ibuprofen	On a case-by-case basis, limited short-term use; monitor total daily dose
	Tramadol	Reduce dose in patients with cirrhosis
	Assess volume status	Hypotension and hypertension may contribute to headache
**Severe headache**	Hydrocodone, oxycodone	Appropriate for in-hospital treatment or short-term outpatient treatment
	Referral to neurology -if titration becomes limited or headache becomes chronic	May be helpful with patients who have a history of migraines
	Evaluate for secondary cause (i.e. check INR, brain imaging, referral to specialist)	Rule out life-threatening cause of acute onset or worsening headache
**Dizziness/ Hypotension**	Decrease blood pressure medication if needed and/or diuretics	Prostacyclins can lower blood pressure
	Assess and managed dehydration or over-diuresis	Diuretics combined with fluid and sodium restriction can occasionally lead to dehydration
	Close monitoring of blood pressure by patient in the home with parameters to call clinic	Establish pattern of low blood pressure
	Caution about sudden change in position	Orthostatic hypotension
	Avoid inadvertent bolus of prostacyclin, increase fluids, decrease dose, vasopressors may be needed	Bolus can lead to hypotension and/or syncope
	Decrease dose if needed	Dose-limiting side effect
	If pre-syncopal or syncope event, evaluate in clinic or hospital	May indicate clinical worsening
**Nausea/Vomiting**	Take with food, eat small frequent meals, ginger-based foods (ginger ale)	
	Anti-emetics: Ondansetron	Vomiting can lead to vagal event and should be avoided
	For inhaled therapies, swish and spit after each treatment session, temporarily decrease by one breath four times a day	Swallowing inhaled prostacyclin can lead to nausea
	Rule out pregnancy	Pregnancy can be life-threatening in PH
	Refer to gastroenterologist	Rule out other causes
	Slow titration or decrease dose	Dose-limiting side effect
**Loss of appetite/ Weight loss**	Dietary consult	Individualized assessment to calculate nutritional needs
	Increase caloric content, small frequent meals, nutritional supplement	Smaller, more frequent meals better tolerated
	Evaluate for other metabolic causes of weight loss	Thyroid dysfunction and cancer can contribute to weight loss
**Diarrhea**	Diphenoxylate/Atropine	
	Loperamide	
	Slow up-titration or decrease dose	Dose-limiting side effect
	Dietary changes: increase fiber, gluten free, low fat, BRAT diet	Decrease motility
	Probiotic	Restore normal bowel flora
	Decrease diuretic	Avoid hypovolemia
	Rule out other causes, such as *C. diff*	
	Refer to GI	Rule out other conditions/complications
**Flushing/Rash**	Reassurance	Reduce anxiety
	Slow down up-titration only if absolutely needed	Not considered a dose-limiting side effect
	Cold packs, compress at back of neck	Relieve feeling of warmth
	Inhaled therapies: if severe, can decrease by one breath four times a day and then increase again when symptom improves	Decrease vasodilation
**Cough**	Inhaled anticholinergics or beta agonists	Relief of bronchospasm
	Inhaled steroids	Reduce inflammation
	Oral phenol-based analgesic sprays administered before treatments	Reduce throat irritation by numbing the throat
	Cough medicine over the counter or prescribed cough preparations but avoiding cough drops to decrease the risk of aspiration	Reduce incidence or intensity of cough
	Drink very cold or warm water before a treatment	Cold for numbing effect; warm for soothing and relaxing effect
	Maintain normal breathing pattern (aerosol is “waiting” for the patient during the green light and they just need to breathe it in)	Improves administration and distribution of medication
	Do not hold breath once medication is inhaled	Not needed for medication administration
	Use the pause button between breaths if needed	When learning, this reduces anxiety associated with the breathing treatment pattern
	Reduce the number of breaths per treatment and/or titrate slower (e.g. by one breath four times a day)	Allows the patient to adapt to the medicine and increases tolerance

GABA, gamma-amino butyric acid; GI, gastrointestinal; H1/H2, histamine; HCl, hydrogen chloride; INR, prothrombin time international normalized ratio; PH, pulmonary hypertension; PLO, pluronic lecithin organogel; LFT, liver function test.

## Pain

Pain is a potential side effect following dosing with all prostacyclin pathway therapies, which can be a significant burden for patients with PAH and can be challenging to manage. Preclinical studies have shown that prostacyclin is a mediator in inflammation and pain and may produce hyperalgesia.^29,30^ Pain may be in the form of jaw pain, leg pain, or generalized body pain. The healthcare provider should assess pain severity and quality, to determine if the pain is typical of a prostacyclin pathway therapy side effect or related to another cause.

Depending on the pain severity, pharmacologic or non-pharmacologic interventions may be appropriate. Non-pharmacologic measures include use of a heating pad, massage, acupuncture, acupressure, and relaxation techniques.^31^ Pharmacologic treatments for pain can include acetaminophen, ibuprofen (if not otherwise contraindicated), gabapentin, pregabalin, tramadol, and narcotics such as hydrocodone, fentanyl, or codeine, on a case-by-case basis.^31,32^ Gabapentin may be a more successful analgesic for leg pain. If the pain is not relieved with the above measures, a lower dose or different prostacyclin therapy may be tried. If the dose is lowered, an attempt can be made to titrate the dose back up once the pain is controlled. In cases of refractory pain, referral to a pain management or palliative care specialist can be considered.^31^

Jaw pain is a side effect unique to prostacyclin pathway therapies and often occurs with the first bite of a meal. It is generally not dose-limiting and can usually be managed by taking slow bites, sucking on a saltine cracker or a hard candy, or chewing gum before eating. Patients should be educated to expect jaw pain.^32^ Myalgia, or muscle pain, has been reported in patients receiving prostacyclin pathway therapies and can be treated as other pain is treated. Additionally, a recent consensus statement regarding treatment of leg pain associated with oral treprostinil recommended screening for iron deficiency.^32^

## Headache

Headache can be a side effect of all of the medications that target the prostacyclin pathway. Prostacyclin has inflammatory and hyperalgesic properties, with actions very similar to those of prostaglandin E1, which has long been known to cause “vascular-type” headaches.^33^ Patients may experience headache of varying severity, quality, frequency, and duration. Best practices exist in the evaluation of headache severity.^34^ Patients with a past history of headache may be more likely to experience headache with dose titration. As is true for all prostacyclin pathway therapy side effects, the rate of dose titration can be slowed to allow for better tolerability. Patients may benefit from scheduled, prophylactic, treatment for headache. For example, before each dose of PO or inhaled prostacyclin pathway therapies or prior to titration of parenteral prostacyclin, patients can be pre-medicated with acetaminophen or other pain medications to attempt to prevent headache onset or reduce severity. If headache is atypical, new, or more severe than expected, consider the possibility of intracranial hemorrhage and check prothrombin time if on warfarin, and consider whether brain imaging or specialist referral is indicated.

## Dizziness and hypotension

Dizziness has been reported in patients who have received epoprostenol and inhaled treprostinil. In a recent consensus statement about dizziness associated with PO treprostinil, the authors suggest closely monitoring blood pressure, lowering doses of blood pressure medicine, and managing fluid volume status.^32^ If dizziness is leading to pre-syncope or syncope, the healthcare provider should thoroughly evaluate the patient in the clinic or hospital setting.

Hypotension has been seen following administration of epoprostenol, iloprost, and SQ and IV treprostinil, but is rarely dose-limiting. There may be an increased rate of hypotension in patients on background anti-hypertensives and on combination PAH therapy compared with monotherapy.^18,21,35^ In patients who are taking anti-hypertensive medications, consider whether the dose should be decreased when prostacyclin pathway therapies are started. Patients should monitor their blood pressure and be advised to change positions slowly and report symptomatic hypotension. An inadvertent bolus of prostacyclin may result in hypotension and patients should make their healthcare provider aware immediately if this occurs. In these instances, a temporary decrease in dose may be required. Severe hypotension may require hospitalization and temporary use of vasopressors.

## Hyperthyroidism and anemia

Thyroid disease is common in patients with PAH and may develop during the disease course.^1^ Prostaglandins may influence thyroid function by a direct effect on specific prostaglandin membrane receptors.^36^ In the selexipag phase 3 clinical trial, new onset hyperthyroidism was reported in eight patients in the selexipag group (1%) and none in the placebo group (*P* = 0.004).^26,35^ Patients with PAH have higher incidence of iron deficiency, cardiac failure, and connective tissue disease complications that predisposes them to anemia.^1^ Anemia was reported in 8% of the selexipag group and 5% of the placebo group (*P* = 0.05).^35^ Baseline medical history of patients in the GRIPHON study,^35^ in which events denoting anemia as previous or concomitant disease were documented in 11.3% and 11.1% of patients randomized to selexipag and placebo, respectively (correspondence with Actelion Pharmaceuticals Inc). Reasons for hyperthyroidism and anemia seen following selexipag treatment are unclear. Serial laboratory follow-up of both measures is recommended for PAH in general.

## Nausea and vomiting

Gastrointestinal complaints have been reported as a cause of decreased quality of life in PAH patients.^37^ Nausea is a common side effect of prostacyclin pathway therapies. If nausea occurs at the time of a dose increase, slower titration or temporarily lowering the dose may help reduce nausea. Non-pharmacologic treatments include: eating smaller, more frequent meals and use of ginger-based products, such as ginger ale.^38^ Anti-emetics can be prescribed to prevent or control nausea. Nausea with inhaled treprostinil may be the result of inadvertent swallowing of the drug, which can be mitigated by rinsing the mouth after each treatment. By selecting the IP receptor, selexipag theoretically has the potential for fewer gastrointestinal side effects, such as nausea and vomiting, that often results from activation of the other prostacyclin receptors by prostacyclins.^39^ Pregnancy should be ruled out in any woman of child-bearing potential when nausea is not clearly a prostacyclin side effect.^24^ If nausea is refractory to the above recommendations, consider referral to a gastrointestinal specialist to evaluate for other causes of nausea.

Loss of appetite is often associated with nausea and can lead to weight loss. In the event of significant weight loss, a dietician consultation should be considered. Some interventions include increasing caloric content, consuming small, more frequent meals, or adding a nutritional supplement. Patients with continued weight loss despite these measures should be evaluated for other causes of weight loss.

## Diarrhea

All prostacyclin pathway therapies can cause diarrhea, which can usually be managed successfully with scheduled, or as-needed doses, of loperamide.^32^ For refractory diarrhea, a slower titration or dose reduction may be necessary until symptom relief is obtained. Dietary changes may be implemented such as adding probiotics or fiber, or switching to a gluten-free diet. If diarrhea is severe, an infection such as *Clostridium difficile* should be excluded. During times of severe diarrhea, the diuretic regimen may need to be reduced to avoid hypovolemia. The healthcare provider should consider referral to a gastroenterologist if the diarrhea is not clearly caused by prostacyclin pathway therapy.

## Flushing

**Fig. 2.**
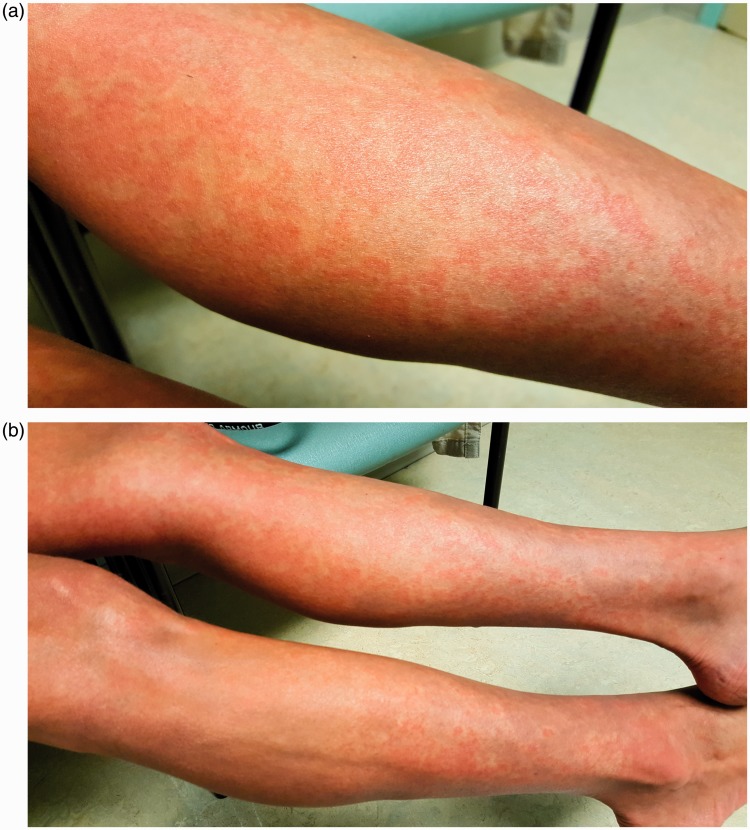
Photos of rash (courtesy of Robin Hohsfield, RN, BSN, University of Colorado Health, Denver, CO, USA; informed consent was received from the patient to include picture in publication).

Vasodilation, or flushing, which may occur after scheduled doses or dose increases, has been observed in patients receiving all of the prostacyclin pathway therapies. Flushing is felt as a warm or hot feeling, sometimes with redness of the skin, which can be generalized or involve only the face. In addition to flushing, patients receiving IV epoprostenol may experience a red rash, which may be constant (Fig. 2).^40^ Fanning or the use of cool packs may be helpful for some patients. Healthcare providers should educate patients about flushing or rash and provide reassurance that it is not harmful and is rarely dose-limiting.^32^

## Cough

Cough and throat irritation with inhaled prostacyclins are most likely related to the route of administration.^24^ Patients with pre-existing lung disease such as interstitial lung disease or chronic obstructive pulmonary disease may experience more cough than patients without lung disease.^24^

For patients who develop cough, the specialty pharmacy staff should review proper technique for administration of inhaled prostacyclins. If coughing persists, treatments include throat lozenges or the use of bronchodilators.^24^

## Thrombocytopenia

Thrombocytopenia has been associated with epoprostenol, iloprost, and parenteral treprostinil,^14,15,18,20^ and has also been associated with more advanced PAH.^41^ Patients with portopulmonary hypertension may have lower platelet counts at baseline and should be followed closely for worsening thrombocytopenia. In rare, severe cases, dose reduction may be necessary or patients could be switched to another PAH therapy. Platelet infusions are rarely necessary. Patients will need routine laboratory testing to monitor for thrombocytopenia and they should report any unusual bleeding.

## Side effects and complications related to route of administration

### Subcutaneous site pain

**Fig. 3.**
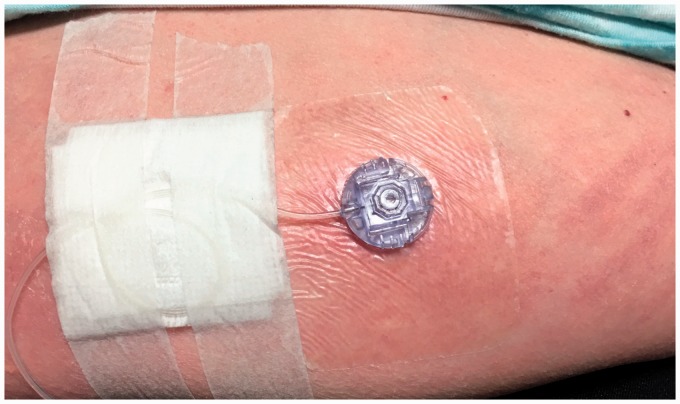
Photo of subcutaneous site reaction (courtesy of Kathy McCloy, NP, University of California, Los Angeles, CA, USA; informed consent was received from the patient to include picture in publication).

Infusion site pain, which is a commonly reported side effect with SQ treprostinil,^31^ typically peaks two to five days after starting a new infusion site and may last up to 14 days.^42^ Pain can be associated with erythema, induration, warmth, inflammation, tenderness, mild site bleeding, nodule, or in severe cases, abscess (Fig. 3).^31^ Site pain varies from patient to patient and can also vary from site to site. Patients require significant support to learn how to manage site pain.^31^ Specific sites for SQ administration include upper buttocks, abdomen, lower flanks, outer thighs, and backs of upper arm. Areas with stretch marks, bruising, edema, or scar tissue should be avoided.^31^

Patients with moderate to severe site pain should be encouraged to maintain sites as long as possible, while monitoring for signs of early infection.^43^ Patients may require several pain control measures sequentially or in combination to reduce site pain such as pluronic lecithin organogel (PLO gel), which is a microemulsion-based gel that has been used to deliver different types of pain drugs topically and transdermally. Additionally, ice packs or lidocaine patches can be used. If site pain has resolved and then recurs days or weeks later, consideration should be made for possible site infection. If site pain is much more severe than usual shortly after placement, consider immediate replacement to another location. Some patients may experience less site pain or reaction with a different catheter type, such as the Cleo® 90 (Smiths Medical, Dublin, OH, USA) or Quick-set® (Medtronic, Northridge, CA, USA), which can be managed by the specialty pharmacy staff. Consider preventative measures prior to site change to reduce pain severity, such as dry catheter pre-placement, treatment with analgesics, amitriptyline HCl, or histamine (H_1_/H_2_) receptors blockers. If needed, gabapentin or tramadol could be administered within the first week of a site change.^31^

## Complications of continuous infusions

### Line infection

Line infections are a known risk for patients receiving intravenous infusions and are a complication of the route of administration, rather than a side effect. Bloodstream infections are most commonly gram-positive infections; however, gram-negative infections have been shown to be higher in patients receiving IV treprostinil than in those receiving IV epoprostenol.^23^ Patients treated with treprostinil with epoprostenol diluent have been shown to have a lower incidence of gram-negative bloodstream infection than those treated with treprostinil with saline diluent and a similar rate to those treated with epoprostenol.^44^ Patients should avoid wetting the infusion system connections,^42^ and a closed-hub system is recommended. Central line infections generally involve removing the infected line and use of a PICC line until the infection has cleared. Peripheral IV lines should be avoided or used for very short durations as they can become painful and are generally too distal from the central circulation. Subcutaneous dosing has a low risk for bacteremia or venous thrombosis, as it does not require central venous access.^19^

## Discussion

Prostacyclin pathway therapies offer significant benefit to patients with PAH in terms of efficacy and survival, but also carry an often difficult-to-manage side effect profile. In order to maintain adherence, it is imperative to carefully manage side effects. At the time the decision is made to initiate a prostacyclin pathway therapy, clinicians should educate patients on what to expect. Most patients will experience some side effects; however, the type and severity will differ from patient to patient. Patients should be counseled to contact their healthcare provider if side effects become too severe, so they can be managed before the need to limit dose increases and to avoid discontinuations. Patients should be reassured that in most cases, side effects will either lessen or abate over time, and the improvement in PAH symptoms will likely outweigh the difficulty faced with side effects during the titration phase.

There are many side effect management strategies that healthcare providers can use to manage prostacyclin side effects. Some approaches will work for one patient and not another; thus, it is important to have a clear understanding of options to combat side effects and tailor these to what works for an individual patient. Patients should be provided clear verbal and written instructions on side effect management. Smart phrases can be created in electronic medical record systems to easily provide detailed side effect management instructions to patients with simultaneous documentation in the medical record. Healthcare professionals should foster open communication and educate patients on side effect plans at the time therapy is started. In particular, nurses are in a key position to provide patient support and counseling, thereby helping patients remain on therapy.

Limitations of this article include the lack of randomized controlled trials in the management of prostacyclin pathway therapy side effects and thus the recommendations are from other disease states as well as the experience of the authors. The similarities in the management of side effects across multiple experienced PH centers adds strength to these recommendations, but evidence-based support is lacking.

## Conclusions

Side effect management following prostacyclin pathway therapy can be challenging for both patients and healthcare providers. By anticipating side effects and initiating a proactive plan, healthcare providers can help patients stay on therapy and receive the maximum benefit from prostacyclin therapy.
